# Defatted Flaxseed Flour as a New Ingredient for Foodstuffs: Comparative Analysis with Whole Flaxseeds and Updated Composition of Cold-Pressed Oil

**DOI:** 10.3390/nu16203482

**Published:** 2024-10-14

**Authors:** Diana Melo Ferreira, Susana Machado, Liliana Espírito Santo, Maria Antónia Nunes, Anabela S. G. Costa, Manuel Álvarez-Ortí, José E. Pardo, Rita C. Alves, Maria Beatriz P. P. Oliveira

**Affiliations:** 1 LAQV/REQUIMTE, Faculty of Pharmacy, University of Porto, Street of Jorge Viterbo Ferreira, 4050-313 Porto, Portugalsu_tche@hotmail.com (S.M.); lilianaespiritosanto81@gmail.com (L.E.S.); antonianunes.maria@gmail.com (M.A.N.); acosta@ff.up.pt (A.S.G.C.); rcalves@ff.up.pt (R.C.A.); 2 Higher Technical School of Agricultural and Forestry Engineering, University of Castilla-La Mancha, Campus Universitario, s/n, 02071 Albacete, Spain; manuel.alvarez@uclm.es (M.Á.-O.); jose.pgonzalez@uclm.es (J.E.P.)

**Keywords:** *Linum usitatissimum* L., food security, nutritional security, functional foods, cold pressing, minimal processing, sustainability, fatty acids, amino acids, antioxidants

## Abstract

Background: Flaxseeds are functional foods popular in current diets. Cold-pressing is a solvent-free method to extract flaxseed oil, resulting in a by-product—defatted flour. Objectives/Methods: This study compared whole flaxseeds and defatted flour (proximate composition, fatty acids, vitamin E, total phenolics and flavonoids, antioxidant activity, amino acids, and protein quality) and updated the composition of cold-pressed oil (oxidative stability, peroxide value, UV absorbance, colour, fatty acids, vitamin E, total phenolics and flavonoids, and antioxidant activity) to assess the nutritional relevance and potential for food applications of these samples. Results: The flour had higher ash (6% vs. 4%), fibre (36% vs. 34%), protein (28% vs. 16%), phenolics (205 vs. 143 mg gallic acid equivalents/100 g), and antioxidant activity than seeds (*p* < 0.05), so it should be valued as a novel high-fibre food ingredient with high-quality plant-based protein, as it contains all essential amino acids (106 mg/g) and a high essential amino acids index (112%), with L-tryptophan as the limiting amino acid. The oil, while low in oxidative stability (1.3 h), due to its high polyunsaturated fatty acids sum (75%), mostly α-linolenic acid (57%), contains a significant amount of vitamin E (444 mg/kg), making it a specialty oil best consumed raw. Conclusions: The exploration of the flour as a minimally processed food ingredient highlights its role in supporting food security, circular economy, and sustainability goals, aligning with consumer preferences for natural, low-fat foods. Future research should investigate the bioactivity and shelf-life of the samples, as well as the bioavailability of compounds after digestion.

## 1. Introduction

The concept of food security includes access to sufficient, affordable, and nutritious food. However, nutritional security also incorporates access, availability, and affordability of foods that are safe, nutritious, align with food preferences, promote well-being, and prevent diseases [1]. Functional foods are foods that provide health benefits beyond basic nutrition, often due to the presence of bioactive compounds. These foods, which can include fortified, enriched, or natural foods, are designed to improve health and reduce the risk of disease. Functional foods play a crucial role in modern diets, as they offer an accessible way to enhance well-being through everyday eating habits [2]. Oilseeds have recently gained popularity due to their bioactive-rich composition, which can meet the global nutritional requirements of a growing population [3]. Examples of both functional foods and oilseeds include chia, sesame, and poppy seeds [4,5,6,7].

Flaxseeds (*Linum usitatissimum* L.), also known as linseeds, are another example. According to FAOSTAT, in 2022, flaxseed was mainly grown in the Russian Federation (almost 1.767 million tonnes), followed by Kazakhstan (almost 846 thousand tonnes), totalling almost 3.974 million tonnes worldwide [8].

Besides their rich nutritional content, flaxseeds have been recognized for their functional food properties, contributing to both human health and environmental sustainability [3,9]. The high levels of lignans (antioxidants) in these seeds are associated with potential anticancer properties, particularly in hormone-related cancers such as breast cancer [9,10]. Moreover, the dietary fibre found in flaxseeds is not only beneficial for digestive health and gut microbiota regulation [3,9] but also for regulating blood sugar levels, making it an important food for managing conditions like diabetes [9,11]. The global emphasis on plant-based diets has further highlighted the importance of flaxseeds as a sustainable source of essential nutrients, particularly in meeting the protein and *n*-3 FA requirements in populations with limited access to animal-based foods [9,12].

Cold-pressing, which relies on mechanical pressing, preserves the natural antioxidants that contribute to oil stability [13]. This is particularly important in oils rich in polyunsaturated fatty acids (PUFA), such as flaxseed oil, to prevent oxidation [14]. The by-products (defatted flours) resulting from oil extraction are generally considered wastes and should be further exploited by the food industry to prevent the depletion of the agricultural sector, while promoting sustainability and increasing food availability [5,6,7]. This approach is a step towards achieving the Sustainable Development Goal (SDG) 2 (zero hunger) [15].

Furthermore, the cold-pressing method is gaining attention not only for preserving the nutritional properties of the oil but also for its low environmental impact, aligning with broader goals of reducing carbon footprints in food production. The valorisation of by-products is a crucial step towards a circular economy, where waste materials are repurposed into valuable food ingredients, thus contributing to food security and sustainability purposes [5,6,7,16].

Recently, consumer demand for natural and minimally processed products with enhanced nutritional and healthy features has motivated cold-pressed oils as a food alternative. However, due to their high market price, they are susceptible to adulteration with cheaper and lower-quality refined oils [13]. Thus, one of the aims of this work was to update the physicochemical composition of cold-pressed flaxseed oil, while also discussing its food applications and potential functional and health-promoting traits. This will help to monitor its authenticity, regarding human health, consumer demand, and quality control.

This work also aimed to perform an overall chemical characterization of whole flaxseeds and their defatted flour (oil extraction by-product) and to compare them to assess their nutritional relevance. Moreover, the flour’s potential as a novel food ingredient was explored, meeting current natural, minimal-processed, and low-fat diets. Also, valorising the flour for food purposes seems an important target to meet food security, nutritional security, circular economy, and sustainability goals. Additionally, an evaluation of the protein quality of the flour and seeds based on the amino acids (AAs) contents was conducted.

## 2. Materials and Methods

### 2.1. Chemicals

The chemicals used in this research were of analytical reagent grade. Boric acid was acquired from Labkem (Barcelona, Spain). Kjeldahl tablets, sodium sulphate, anhydrous sodium sulphate, and Folin–Ciocalteu reagent were acquired from Merck (Darmstadt, Germany). Sulfuric acid and methanol were from Honeywell Fluka^TM^ (Düsseldorf, Germany). Petroleum ether and hydrochloric acid (1 M) were provided by CARLO ERBA Reagents (Val de Reuil Cedex, France). Sand, sodium hydroxide (1 M), and sodium dihydrogen phosphate were provided by VWR (Strasbourg, France). Ethanol (96%) was from AGA (Prior Velho, Portugal). Acetic acid (99–100%) was provided from Chem-Lab (Zedelgem, Belgium). A standard mixture (FAME 37) was obtained from Supelco (Bellefonte, PA, USA). Total dietary fibre assay kit, celite, sodium carbonate, gallic acid, sodium nitrate, aluminium chloride, sodium acetate, ferric chloride, TPTZ (10 mM), ferrous sulphate, catechin, and HPLC-grade solvents were acquired from Sigma-Aldrich (St. Louis, MO, USA). Vitamin E standards (α, β, δ, γ-tocopherols and tocotrienols) were obtained from Calbiochem (La Jolla, CA, USA). The amino acids standards: L-aspartic acid (Asp, ≥99.5%), L-glutamic acid (Glu, ≥99.0%), L-asparagine (Asn, ≥99.0%), L-glutamine (Gln, ≥99.5%), L-alanine (Ala, ≥99.5%), L-arginine monohydrochloride (Arg, ≥99.0%), L-cystine (Cys, ≥99.0%), L-valine (Val, ≥99.0%), L-threonine (Thr, ≥99.0%), L-tyrosine (Tyr, ≥98.0%), L-leucine (Leu, ≥98.0%), L-tryptophan (Trp, ≥98.0%), L-lysine monohydrochloride (Lys, ≥99.0%), glycine (Gly, ≥99.0%), L-phenylalanine (Phe, ≥98.0%), L-serine (Ser, ≥99.0%), L-methionine (Met, ≥99.0%), L-isoleucine (Ile, ≥99.0%), trans-4-hydroxy-L-proline (Hyp, ≥99.0%), L-proline (Pro, ≥99.0%), and L-histidine mono-hydrochloride monohydrate (His, ≥99.0%) were purchased from Sigma-Aldrich (Darmstadt, Germany). Ultrapure water was obtained in a Milli-Q water purification system (Millipore, Bedford, MA, USA).

### 2.2. Samples and Sample Preparation

**Figure 1:**
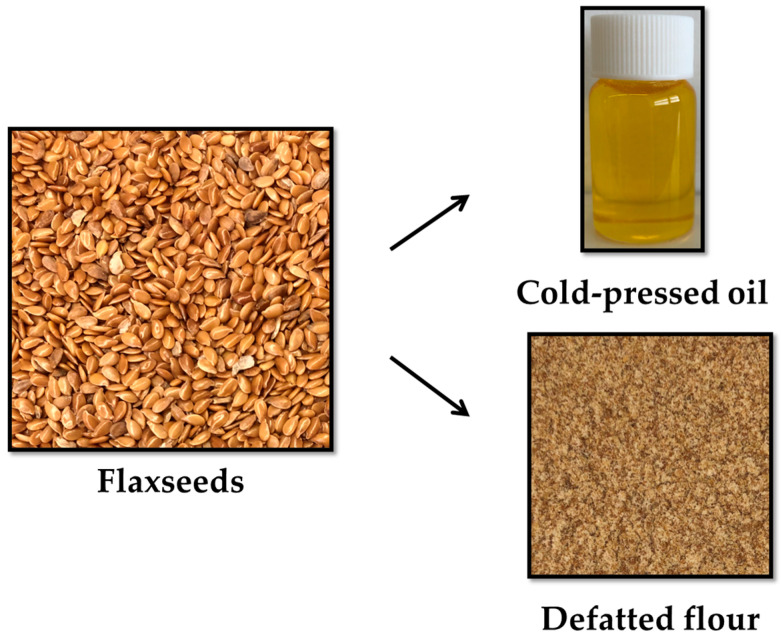
Analysed samples—golden flaxseeds, cold-pressed oil, and defatted flour.

Golden flaxseeds were purchased in a Spanish supermarket. Oil and flour were obtained by cold-pressing 1 kg of flaxseeds using a screw press (Komet Oil Press CA59G, IBG Monforts Oekotec GmbH & Co. KG, Mönchengladbach, Germany). For the oil extraction, a nozzle with a 5 mm diameter was used. The barrel was preheated with a thermal ring until it reached 100 °C. Once the oil extraction process began, the thermal ring was removed, and extraction continued at room temperature (RT). The by-product obtained from cold-pressing the flaxseeds in the screw press was ground for homogenization (GM Grindomix 200, Retsch GmbH, Retsch-Allee, Haan, Germany) and used as the flour sample. The analyses began immediately after sample preparation. The oil was stored in the dark at 4 °C, while the flour and seeds were vacuum-sealed and kept at 4 °C in between analyses to minimize potential oxidative changes. The analysed samples are shown in Figure 1.

### 2.3. Proximate Composition and Energy Values of Whole Flaxseeds and Defatted Flour

The proximate composition of the samples was determined using AOAC standard methods [17]. The moisture content was determined by drying (105 °C), using an infrared balance (model SMO01, Scaltec Instruments, GmbH, Goettingen, Germany). The ash content was determined by sample incineration (500 °C, method no. 920.153), using a muffle furnace (Thermolyne 48000, Thermo Fisher Scientific, Waltham, MA, USA). Total protein was calculated from the nitrogen content by Kjeldahl procedure (no. 978.04) with a nitrogen conversion factor of 6.25 [18], using a Buchi Digestion Unit K-424, a Scrubber B-414, and a KjelFlex K-360 (VELP Scientific, Usmate Velate, MB, Italy). Total fat was determined using a Soxhlet apparatus (method no. 991.36), a Huber Minichiller, and a P Selecta heating mantle (VELP Scientific, Usmate Velate, MB, Italy). Total dietary fibre and insoluble fibre were determined by enzymatic-gravimetric methods (no. 985.29), using a Multistirrer 6, a heating magnetic stirrer, a heated circulating bath, and a CSF 6 filtration system (VELP Scientific, Usmate Velate, MB, Italy). Soluble fibre and remaining carbohydrates were calculated by difference [18]. Energy values were calculated using the following estimates: fibre (2 kcal/g, 8 kJ/g), carbohydrates and protein (4 kcal/g, 17 kJ/g), and fat (9 kcal/g, 37 kJ/g) [19].

### 2.4. Fatty Acids (FA) of All Samples

The lipid fractions of the samples were extracted following the method described by Ferreira et al. [16]. FA were derivatized to fatty acids methyl esters (FAMEs) in accordance with ISO 12966-2:2017 [20]. FA were determined using a GC-2010 Plus gas chromatograph equipped with an AOC-20i automatic sampler and a split/splitless auto-injector (50:1 split ratio, 250 °C) and a flame ionization detector (270 °C) (Shimadzu, Tokyo, Japan). A CP-Sil 88 silica capillary column (50 × 250 m, 0.2 μm) (Varian, Middelburg, The Netherlands) was used for peak separation. Helium was the carrier gas (3.0 mL/min). The temperature programme was 120 °C held for 5 min, 2 °C/min to 160 °C held for 2 min, and 2 °C/min to 220 °C held for 10 min. The injection volume was 1.0 μL. Identification was achieved by comparison with a FAME 37 standard mix (Supelco, Bellefonte, PA, USA).

### 2.5. Vitamin E of All Samples

The lipid fractions of the samples were extracted following the method described by Ferreira et al. [16]. Vitamin E contents were determined using a HPLC system equipped with a MD-2015 multiwavelength diode array detector and a FP-2020 fluorescence detector (excitation at 290 nm and emission at 330 nm) (Jasco, Tokyo, Japan). A Supelcosil^TM^ LC-SI column (75 × 3 mm, 3 μm) (Supelco, Bellefonte, PA, USA) was used for separation. The mobile phase consisted of 1.5% 1,4-dioxane in *n*-hexane (*v*/*v*) at a flow rate of 0.7 mL/min. The injection volume was 20 μL. Tocol (100 μg/mL, Matreya Inc., State College, PA, USA) was used as the internal standard.

### 2.6. Amino Acids (AAs) of Whole Flaxseeds and Defatted Flour

AA extraction and analysis were performed, according to Machado et al. [21], using a HPLC system equipped with MD-2015 Plus multiwavelength and FP-2020 Plus fluorescence detectors (Jasco, Tokyo, Japan) and a ZORBAX Eclipse Plus C18 column (40 °C, 4.6 × 250 mm, 5 μm, Agilent Technologies, Santa Clara, CA, USA). Alkaline (KOH 4M, 4 h, only for Trp) and acid hydrolysis (HCl 6M, 24 h) were performed in glass tubes using a heating block (110 °C, SBH130D/3, Stuart, Stafford, UK). Aliquots of hydrolysates were mixed with internal standard (L-norvaline, 2 mg/mL, Sigma-Aldrich, Darmstadt, Germany), and the mixtures were subjected to automatic online derivatization with OPA/3-MPA and FMOC in a AS-4150 autosampler. Fluorescence detection was monitored at λ_exc_ = 340 nm and λ_em_ = 450 nm (0–26.2 min) for OPA-derivatives and at λ_exc_ = 266 nm and λ_em_ = 305 nm (26.2–40 min) for FMOC-derivatives. OPA-derivatives were monitored at 338 nm and FMOC-derivatives at 262 nm. The gradient solvent system was (A) phosphate/borate buffer (ratio of 10 mM of Na_2_HPO_4_ to 10 mM of Na_2_B_2_O_7_ [pH = 8.2] to 5 of mM NaN_3_) and (B) MeOH:ACN:H_2_O (45:45:10, *v*/*v*/*v*). The gradient program was as follows: 0.85 min, 2% B; 33.4 min, 57% B; 33.5 min, 85% B; 39.3 min, 85% B; 39.4 min, 2% B; and 40.0 min, 2% B. The flow rate was 1.5 mL/min. The injection volume was 3 μL.

### 2.7. Protein Quality of Whole Flaxseeds and Defatted Flour

The following equations were used to evaluate protein quality [22,23]:

#### 2.7.1. Estimation of Amino Acid Chemical Score (AACS)


(1) $$AACS\left(\%\right)=\frac{mg\;of\;AA\;in\;1\;g\;test\;protein}{mg\;of\;AA\;in\;1\;g\;requirement\;protein}\times 100$$

AA—amino acid.

#### 2.7.2. Estimation of Essential Amino Acids Index (EAAI)


(2) $$EAAI\left(\%\right)=n^{log\;EAA},\;where\;\log EAA=\frac{1}{n}\left(log\frac{100\;a1}{a1R}+\ldots +log\frac{100\;an}{anR}\right)$$

EAA—essential amino acid; n—number of amino acids (methionine and cysteine pairs count as 1); a—mg of amino acid per 1 g of protein in the food; and aR—mg of amino acid per 1 g of reference protein.

### 2.8. Total Phenolics and Total Flavonoids of All Samples

The extracts were prepared following the methods described by Melo et al. [5].

Total phenolics were determined according to Ferreira et al. [7]. In brief, 30 µL of each extract were mixed with 150 µL of Folin–Ciocalteu reagent and 120 µL of sodium carbonate (7.5%, m/V). The microplate was incubated at 45 °C (15 min), then cooled and protected from light (RT, 30 min). Absorbance was read at 765 nm using a microplate reader (Synergy HT GENS5, BioTek Instruments, Winooski, VT, USA). A calibration curve was generated with gallic acid (5–100 mg/mL, R^2^ = 0.999). Results are expressed as gallic acid equivalents (GAEs).

Total flavonoids were also determined according to Ferreira et al. [7]. Briefly, 1 mL of each extract was mixed with 4 mL of distilled water and 300 µL of sodium nitrate (1%). After 5 min, 300 µL of aluminium chloride (5%) were added. After 1 min, 2 mL of sodium hydroxide (1 M) and 2.5 µL of distilled water were added. Absorbance was measured at 510 nm using a microplate reader (Synergy HT GENS5, BioTek Instruments, Winooski, VT, USA). A calibration curve was generated with epicatechin (0–400 µL/mL, R^2^ = 0.999). Results are expressed as epicatechin equivalents (EE).

### 2.9. Ferric Reducing Antioxidant Power (FRAP) of All Samples

The extracts were prepared according to Melo et al. [5]. FRAP assay was determined according to Ferreira et al. [7]. In a microplate, 35 µL of each extract were mixed with 265 µL of FRAP reagent (0.3 M acetate buffer, 10 mM TPTZ solution, and 20 mM of ferric chloride) and incubated protected from light (37 °C, 30 min). Absorbance was measured at 595 nm. A calibration curve was prepared with ferrous sulphate (25–500 μmol/L, R^2^ = 0.999). Results are expressed as ferrous sulphate equivalents (FSEs).

### 2.10. 2,2-Diphenyl-1-picrylhydrazyl Radical (DPPH^•^) Inhibition of All Samples

The extracts were prepared according to Melo et al. [5]. DPPH^•^ inhibition assay was evaluated according to Ferreira et al. [7]. In a microplate, 30 µL of each extract were mixed with 270 µL of an ethanolic solution of DPPH^•^. Absorbance was measured at 525 nm, every 2 min, until 20 min, to observe the kinetics reaction. A calibration curve was prepared with Trolox (5.62–175.34 mg/L, R^2^ = 0.998). Results are expressed as Trolox equivalents (TE).

### 2.11. Oxidative Stability of Cold-Pressed Flaxseed Oil

The oxidative stability was determined using the Rancimat method as described by Melo et al. [5]. Briefly, 3 g of oil were analysed, at 120 °C with an airflow rate of 20 L/h using a Rancimat apparatus (model 892, Metrohm Nordic ApS, Glostrup, Denmark). Results are expressed as oxidation induction time (h).

### 2.12. Peroxide Value of Cold-Pressed Flaxseed Oil

The peroxide value was determined according to NP-904:1987 [24]. Briefly, 0.5 g of sample, 10 mL of chloroform, 15 mL of glacial acetic acid, and 1 mL of saturated KI solution were mixed and stored in the dark (5 min). After that, 75 mL of deionized water were added, followed by titration with 0.01 N sodium thiosulfate and 1% starch solution.

### 2.13. UV Absorbance of Cold-Pressed Flaxseed Oil

The UV absorbance was determined following ISO 3656:2011 [25]. Primary and secondary oxidation products were read at 232 and 270 nm, respectively, using a Shimadzu UV Spectrophotometer UV-1800 (Shimadzu, Tokyo, Japan).

### 2.14. Colour of Cold-Pressed Flaxseed Oil

The colour was determined according to NP-937:1987 [26], at 445, 495, 560, 595, and 625 nm, using a Shimadzu UV Spectrophotometer UV-1800 (Shimadzu, Tokyo, Japan).

### 2.15. Statistical Analysis

Data were analysed with IBM SPSS Statistics (version 28, IBM Corp., Armonk, NY, USA). An independent samples *t*-test was applied to reveal significant differences between seeds and flour samples, with a 95% interval of confidence (*p* < 0.05). All determinations were performed in triplicate (*n* = 3).

## 3. Results

### 3.1. Comparative Analysis of Whole Flaxseeds and Defatted Flour

The results of the composition of flaxseeds and flour are presented in Table 1.

The results from Table 1 indicate significant differences in the nutritional composition of whole flaxseeds and defatted flour. The energy value of whole seeds was higher, with 475 kcal/100 g (1954 kJ/100 g) compared to the flour’s 314 kcal/100 g (1304 kJ/100 g). The flour had a slightly higher moisture content at 9.8% compared to the seeds’ 8.4%. The ash content was also greater in the flour (5.6%) than in the seeds (3.6%). The flour had a higher protein content (28.1% vs. 16.1%) and total dietary fibre (35.6% vs. 33.7%), with more soluble fibre (10.0%) but less insoluble fibre (25.7%) compared to the seeds (3.5% soluble and 30.2% insoluble). The fat content was significantly reduced in the flour (9.3%) compared to the seeds (38.1%).

In terms of FA (Table 1), the flour showed higher percentages of palmitic acid (6.3% vs. 5.1%) and linoleic acid (17.1% vs. 16.6%), but a lower concentration of α-linolenic acid (54.9% vs. 56.8%). The flour also contained significantly less total vitamin E (48.5 mg/kg) than the whole seeds (214.7 mg/kg), along with lower levels of α-tocopherol (17.4 mg/kg vs. 73.7 mg/kg) and γ-tocopherol (31.0 mg/kg vs. 141.0 mg/kg).

However, the total AA content was much higher in the flour, with 314.2 mg/g compared to 194.2 mg/g in the seeds. Each AA, including essential ones like Lys (16.0 mg/g vs. 9.1 mg/g), Leu (18.8 mg/g vs. 11.8 mg/g), and Phe (15.9 mg/g vs. 9.8 mg/g), was more concentrated in the flour. Additionally, the flour had higher contents of total phenolics (205 mg GAE/100 g vs. 143 mg GAE/100 g) and total flavonoids (78 mg EE/100 g vs. 54 mg EE/100 g), and exhibited greater antioxidant activity, as indicated by FRAP (14 mmol FSE/100 g vs. 9 mmol FSE/100 g) and DPPH^•^ inhibition (233 mg TE/100 g vs. 57 mg TE/100 g) assays.

Overall, these results suggest that defatted flaxseed flour is a nutrient-dense ingredient, rich in protein, AAs, fibre, and antioxidants, making it a potentially valuable component for various food applications.

The results of the protein quality assessment are displayed in Table 2. Whole flaxseeds exhibited significantly higher levels of His, Ile, Leu, Phe + Tyr, Thr, and Trp compared to the flour. However, Met and Val levels did not show significant differences. Specifically, His content was 38.8 mg/g protein in seeds vs. 34.0 mg/g protein in flour, with corresponding AACSs of 258.7% and 226.9%, respectively. Ile was found at 53.1 mg/g protein in seeds compared to 46.1 mg/g protein in flour, yielding AACSs of 177.2% and 153.6%, respectively. Leu levels were 73.6 mg/g protein in seeds and 66.8 mg/g protein in flour, with AACSs of 124.8% and 113.3%, respectively. Lys content was nearly identical between seeds (56.8 mg/g protein) and flour (56.9 mg/g protein), and the AACSs were also comparable at 126.1% and 126.4%, respectively. Met levels were higher in seeds (17.4 mg/g protein) than in flour (14.1 mg/g protein), with AACSs of 109.0% and 88.0%, respectively. For Phe + Tyr, seeds had a content of 87.7 mg/g protein vs. 79.4 mg/g protein in flour, with AACSs of 230.7% and 209.0%, respectively. Thr levels were 48.2 mg/g protein in seeds compared to 43.3 mg/g protein in flour, with AACSs of 209.6% and 188.1%, respectively. Trp was significantly higher in seeds (8.7 mg/g protein) compared to flour (5.3 mg/g protein), with AACSs of 144.7% and 87.5%, respectively. Finally, Val content in seeds was 59.4 mg/g protein vs. 54.9 mg/g protein in flour, with AACSs of 152.4% and 140.7%, respectively. The limiting amino acid (LAA) was Met (109.0%) in seeds and Trp (87.5%) in flour. The EAAI was higher in seeds (129.5%) compared to the flour (111.8%).

### 3.2. Cold-Pressed Flaxseed Oil

The results of the composition of the oil are presented in Table 3. The oxidative stability of the oil was measured at 1.3 h, indicating a relatively low resistance to oxidation. The peroxide value, a measure of primary oxidation products, was 2.4 meq O_2_/kg, reflecting minimal initial oxidation. The oil exhibited low absorbance values at 232 nm (K_232_ = 0.02) and 270 nm (K_270_ = 0.05), which are indicative of minimal conjugated dienes and trienes, respectively. Chromatic coordinates were recorded as (x, y) = (0.475, 0.480), with a transparency of 65.8%. The dominant wavelength of 578 nm suggests a yellowish hue, typical for oils with a moderate level of pigmentation.

The FA profile of the oil (Table 3) shows a predominance of PUFAs, which constitute 73.5% of the total FA. The primary FAs include α-linolenic acid at 57.5%, oleic acid at 18.5%, and linoleic acid at 16.0%. The SFAs totalized 8.0%, while MUFAs account for 18.5%. The *n*-6/*n*-3 ratio is 0.3, indicating a higher concentration of *n*-3 FAs compared to *n*-6.

The oil contains a total vitamin E content of 443.9 mg/kg (Table 3). This includes α-tocopherol at 4.0 mg/kg, α-tocotrienol at 3.7 mg/kg, γ-tocopherol at 431.7 mg/kg, and δ-tocopherol at 4.6 mg/kg. Therefore, the predominant form of vitamin E in this oil is γ-tocopherol, which constitutes the majority of the total vitamin E content.

The oil’s phenolics total was 2.3 mg GAE/100 g, while total flavonoids were found to be low (0.4 mg EE/100 g). The antioxidant activity, as assessed by the FRAP assay, was 96.0 μmol FSE/100 g. The DPPH^•^ inhibition assay revealed a low antioxidant activity of 0.2 mg TE/100 g (Table 3).

## 4. Discussion

### 4.1. Comparative Analysis of Whole Flaxseeds and Defatted Flour

Energy used for metabolic homeostasis, thermoregulation, physical activity, and normal organ function is obtained from the oxidation of macronutrients [27].

The present study (Table 1) showed that flaxseeds revealed a significantly higher energy value than the flour (475 vs. 314 kcal/100 g, respectively) due to higher total fat content (38% vs. 9%, respectively) since this is the most energy dense macronutrient (9 kcal/g) [19]. Thus, flaxseed flour seems an interesting choice as a low-fat ingredient for the food industry, which will match current dietary trends focused on weight management and reduced fat intake.

Minerals are nutrients which sustain health, most of them function as cofactors for enzymes, biochemical substrates, and hormones [27].

Regarding the results of the proximate composition (Table 1), total ash content was significantly higher in flaxseed flour in comparison to flaxseeds (6% vs. 4%, respectively, Table 1). Another study reported the contents of individual minerals in flaxseed: K (831 mg/100 g), P (622 mg/100 g), Mg (431 mg/100 g), and Ca (236 mg/100 g) [28]. Thus, the flour becomes a higher source of total minerals in comparison to whole flaxseeds. It has been previously reported that the contents of Mg may help to enhance antioxidant capacity, while K and Ca contents may help to control high blood pressure [4].

The high-fibre contents (36% vs. 34% in flour and seeds, respectively, Table 1) besides providing nutritional value, can also provide gelling properties (e.g., water-holding and swelling capacity), functioning as a thickening and emulsifying agent—properties that can be helpful in developing new foodstuffs. Fibre ingestion can also help reduce glucose and cholesterol blood levels, ultimately decreasing the risk of heart disease and colon cancer. Moreover, flaxseed’s fibre consumption has been associated with hunger suppression. Fibre also functions as a prebiotic stimulating the gut microbiota into producing small-chain FA (e.g., acetate, propionate, and butyrate), which can modulate important metabolic pathways in the organism [28,29,30].

Another study reported a higher fibre content in flaxseeds (soluble fibre at 10% and insoluble fibre at 30%, totalling 40% of dietary fibre), but the total fat content was similar (38.7%), even though moisture content was slightly lower (7%) [31].

Another research reported that flaxseed’s fibre is present mostly in the hull, its soluble fibre was composed of acidic polysaccharides such as L-rhamnose (25%), L-galactose (12%), L-fructose (8%), and D-xylose (29%) and neutral polysaccharides such as L-arabinose (20%) and D-xylose-D-galactose (76%), while its insoluble fibre comprised cellulose (7–11%), lignin (2–7%), and acid detergent fibre (10–14%) [30].

Triglycerides are the main constituents of body fat, when FA are needed as an energy source the bound between the three FA and the glycerol is hydrolysed, the first are released into the blood stream and bound to albumin as free FA [27].

Flaxseeds presented a rich composition in fat, nevertheless cold-pressing the seeds revealed to be an effective method to reduce this high amount (seeds, 38%, vs. flour, 9%; Table 1). The obtained oil can have further applications, e.g., as a dietary supplement, as discussed below.

There are three main families of unsaturated FA—*n*-3, *n*-6, and *n*-9—classified according to the position of their first double bond, e.g., linoleic acid (LA, C18:2n6*c*, two double bonds) and arachidonic acid (C20:4n6*c*, four double bonds) are essential FA and precursors of compounds responsible for inflammation, coagulation, smooth muscle vasoconstriction, and vasodilation [27].

Diet-related noncommunicable diseases (NCDs), e.g., cardiovascular diseases (CVDs), diabetes, and cancer, are associated with major risk factors such as high ingestion of saturated fats (mostly from animal fats), leading to increased low-density lipoprotein (LDL) levels. Alternatively, unsaturated FA particularly oleic acid (OA, C18:1n9*c*), LA (C18:2n6*c*), and α-linolenic acid (ALA, C18:3n3*c*) are associated with NCD prevention [32].

Regarding the FAs profile (Table 1), flaxseeds and flour presented mostly ALA (57% vs. 55%, respectively), followed by OA (18%), and LA (17%), totalling high PUFA contents (seeds, 73%, vs. flour, 72%). These results are similar to those previously reported in another work: saturated fatty acids (SFAs, 8.9–12.7%), monounsaturated fatty acids (MUFAs, 16.1–20.5%), and PUFAs (69.0–73.7%) [32].

ALA (55–57%, Table 1) consumption was associated with lowering triglyceride and cholesterol levels, and blood pressure, as well as anti-inflammatory, antidiabetic, cardiac, and hepatic protective activities by redistributing lipids away from visceral fat and the liver. LA (17%, Table 1) can present inflammatory, hypertensive, and thrombotic activities if present in excessive quantity. A balanced *n*-6/*n*-3 ratio is vital for health and flaxseed samples’ ratio was low (0.3, Table 1) which can compensate for the harmful effects of higher dietary consumption of SFA (in the present samples this sum was low: 8.6–9.7%; Table 1). However, in appropriate levels, LA has an important role in the formation of prostaglandins, leukotrienes, and thromboxane, which are key compounds in several processes in the organism, such as those previously mentioned [4,27].

Vitamin E is a liposoluble antioxidant which protects unsaturated fat from oxidation, especially in seeds. In humans, it presents anti-inflammatory activity, the prevention of neoplastic transformation, the protection of artery walls, and the prevention of LDL oxidation [33]. Particularly, α-tocopherol is a good chain-breaking and peroxyl radical scavenger (seeds, 74 mg/kg, vs. flour, 17 mg/kg; Table 1). γ-Tocopherol (seeds, 141 mg/kg, vs. flour, 31 mg/kg; Table 1) is more active against reactive nitrogen species [33]. Both vitamers were identified in flaxseed samples, γ-tocopherol was the predominant isomer, but seeds presented significantly more total vitamin E than the flour (215 mg/kg vs. 48 mg/kg, respectively; Table 1), probably due to their higher fat content (38% vs. 9%, respectively; Table 1) since this vitamin is liposoluble.

Unlike a previous study [34], the seeds and flour of this work did not present δ-tocopherol. However, as will be discussed later, the oil presented this isomer, but different conditions were applied to extract vitamin E, which could explain the different findings.

Proteins are constituted by chains of AAs linked by peptide bonds. AAs can be D- or L-isomers, with the latter being the naturally occurring form in proteins. There are 20 different AAs found in human proteins. Some are essential and must be obtained in the diet, while others are synthesized by the body (nonessential AAs). Some AAs play other roles besides protein structure, e.g., Tyr (4–6 mg/g, Table 1) is implicated in the formation of thyroid hormones, and Glu (40–65 mg/g, Table 1) is used in the synthesis of neurotransmitters [27].

Flaxseed flour presented a significantly higher total protein content (28%) in comparison to flaxseeds (16%), which was also reflected in the total AA content (314 vs. 194 mg/g, respectively; Table 1). The major AAs identified in the flour and seeds were Glu (65 vs. 40 mg/g, respectively; Table 1), Arg (37 vs. 23 mg/g, respectively; Table 1), and Asp (31 vs. 18 mg/g, respectively; Table 1). Glu was also a major AAs in other studies [31,34]. However, unlike reported by those authors, in the present study, Cys was not identified [31,34].

Recent data about Gln suggest that it plays a key role in cardiovascular health and CVD prevention. Indeed, this was the major AA found in the present samples (Table 1). This AA is a substrate for DNA, ATP, proteins, and lipid synthesis in vascular cells, influencing important processes such as proliferation, migration, and apoptosis. Moreover, it also presents potent antioxidant and anti-inflammatory activities in the circulation by promoting the expression of heme oxygenase-1, heat shock proteins, and glutathione. It is also an Arg precursor in nitric oxide synthesis, having an impact on several NCDs (e.g., hypertension, hyperlipidemia, glucose intolerance, obesity, and diabetes) [35].

Regarding the present results, all essential AAs (His, Thr, Val, Met, Trp, Phe, Ile, Leu, and Lys) were identified, totalling 106 vs. 67 mg/g in flaxseeds and flour, respectively (Table 1). Therefore, all branched-chain AAs (Val, Leu, and Ile) were present, totalling 47 vs. 30 mg/g, respectively (Table 1).

Regarding protein quality (Table 2), the AACS revealed that the LAA (which is the AA with lowest AACS) of flaxseeds was L-methionine (109%), and in the flour, it was L-tryptophan (87%). When comparing both samples, the scores were higher for the seeds in relation to the flour, except for Lys, Met, Phe + Tyr, and Val, which did not exhibit differences (*p* < 0.05). The EAAI was 129% for flaxseeds and 112% for the flour, revealing that both present a high-quality protein since the EAAI was higher than 90% [6], although the seeds presented a significantly higher index than the flour (*p* < 0.05). Overall, flaxseed flour’s protein seems a cheaper source of high-quality protein in comparison to animal protein.

Phenolics are compounds with antioxidant activity which prevent and delay food deterioration, also helping to maintain their quality and nutritional value. In body tissues, they help to prevent damage from oxidative stress [5].

Flaxseeds are a natural source of phenolic compounds. Besides sesame seeds, flaxseeds are one of the major natural sources of lignans. These compounds are antioxidants known for their antidiabetic, antihypertensive, anti-inflammatory, and neuroprotective properties. This seed is the richest source of lignin precursors with possible biological benefits in reducing degenerative disease risk and opposing the lack of oestrogens. Moreover, lignans have been described as anti-carcinogenic compounds. In combination with their ALA content (55–57%, Table 1), these seeds may confer health benefits while allowing chemo-protection. Lignans’ health benefits include lower CVD, osteoporosis, and breast cancer risk. After consumption, plant lignans are metabolized by gut bacteria into mammalian lignans that could prevent cancer [36,37,38].

Flaxseed flour was significantly richer in total phenolics in comparison to whole flaxseeds (205 mg of GAE/100 g vs. 142 mg of GAE/100 g, respectively; Table 1) which also occurred in total flavonoids (78.2 mg of EE/100 g vs. 53.9 mg of EE/100 g, respectively; Table 1). There were also significant differences in the FRAP assay (flour, 14.3 mmol FSE/100 g, vs. seeds, 8.5 mmol FSE/100 g; Table 1) and in the DPPH^∙^ inhibition assay (flour, 233.3 mg TE/100 g, vs. seeds, 57.2 mg TE/100 g; Table 1). Like macronutrient contents, phytochemical yields can vary due to different crops origins, plant genetics, and edaphoclimatic conditions [37].

Interestingly, the flour even with less oil displayed higher contents of the analysed phytochemicals and antioxidant activity, suggesting that these are probably present in flaxseeds’ hulls, possibly as a protective response against environmental factors such as oxygen exposure. Therefore, it seems that the major protector of the oil is probably vitamin E (results discussed below).

Indeed, Bekhit et al. [33] also reported higher contents of total phenolic acids in the defatted extract (8440 mg/100 g) in comparison to the non-defatted extract (2767 mg/100 g), mainly *p*-hydroxybenzoic acid, followed by ferulic acid, coumaric acid, gallic acid, vanillin, sinapic acid, protocatechuic acid, and caffeic acid.

Another study obtained methanolic extracts from flaxseeds and flaxseed meal (2.7% fat), and similarly to the present findings, the obtained results were higher for the defatted meal. Total phenolics contents were 1538.7 μg/g for seeds and 1728.1 μg/g for the meal. Total flavonoids contents were 1.7 mg/g for seeds and 2.8 mg/g for the meal. FRAP assay results were 0.13 mmol TE/g for seeds and 1.09 mmol TE/g for the meal. DPPH^•^ assay results were 0.12 mmol TE/g for seeds and 0.42 mmol TE/g for the meal [39].

Deme et al. [32] also analysed total phenolics (20.5–25.4 mg GAE/g) and FRAP (15.3–30.6 mmol Fe_2_SO_4_/100 g), but different extracting conditions were used (ethanol and ultrasonic bath), impairing direct comparisons with our results.

The bioavailability of the studied compounds after processing, cooking, and digestion should be evaluated in further studies.

When comparing the nutritional profiles of flaxseeds and their respective defatted flour (Table 1) with other popular seeds—sesame, poppy, and chia—notable differences emerge in the contents of macronutrients.

Sesame contains higher levels of ash, with 5% in seeds and 7% in cake, indicating greater mineral content. Additionally, sesame has slightly higher protein levels (19% in seeds and 30% in cake) and also has a higher fat content (53% in seeds and 32% in cake) than flax. However, regarding dietary fibre, flaxseeds excel, surpassing sesame which contains 20% in seeds and 25% in cake. Overall, while flax is richer in fibre, sesame offers more fat and minerals [5].

Poppy has an ash content of 7% in seeds and 10% in cake, indicating a higher mineral content, but contains lower protein levels (15% in seeds and 26% in cake) than flax. Poppy and flax present similar fat contents (39% in poppy seeds and 10% in poppy cake). Regarding dietary fibre, poppy contains lower levels (32% in seeds and 38% in cake). In summary, while flax tends to have higher fibre and protein contents, poppy is richer in minerals [6].

Chia has an ash content of 5% in seeds and 6% in cake, whereas flax exhibits lower ash levels. Both seeds are comparable in terms of protein (chia contains 18% in seeds and 27% in cake). Chia has a total fat content of 33% in seeds and 7% in cake. This indicates that flax is richer in fat compared to chia seeds. Regarding dietary fibre, chia seeds excel with a total dietary fibre content of 38% in seeds and 48% in cake, surpassing flaxseeds, which have 34% in seeds and 36% in flour. On the whole, while chia provides higher fibre and mineral contents, flax is richer in fat [7].

All of these seeds are in-demand in current diets because they have similar food applications and can provide several health benefits when consumed [4,5,6,7].

### 4.2. Defatted Flaxseed Flour as a New Ingredient for Foodstuffs

Defatted flaxseed flour provides new food options to consumers and manufacturers as a high-value by-product from oilseeds processing. It has potential for the R&D of new foodstuffs since it is a natural, minimally processed, functional ingredient, rich in dietary fibre and high-quality gluten-free protein [5,6,7]. It also contributes to the prevention of NCD because it is a low-fat ingredient since a high energy-dense and ultra-processed diet is known to induce obesity [40,41,42].

This by-product can be incorporated in novel foods as a functional ingredient such as bakery products, e.g., flaxseed flour (7.5%), was useful in producing fortified sourdough bread [43] and defatted flaxseed meal (5–10%) was used in toast and cake to partially substitute the wheat flour [44]. The use of this by-product for food purposes ensures food and nutritional security due to its complete composition in bioactive compounds, also promoting environmental sustainability with a zero-waste approach [1,45]. This approach also aligns with current food concerns, in particular to meet the SDG 2 [15].

Another study reported higher contents of total protein (35%) and fibre (37%) for flaxseed flour in relation to the present results, probably because of the lower total fat (2%) and moisture contents (6%) when compared to the present data. However, ash content was similar (6%). Like the present data, extracts showed a strong antioxidant activity against DPPH^•^ [46].

### 4.3. Cold-Pressed Flaxseed Oil

Cold-pressed flaxseed oil presented a low oxidative stability (1.3 h, Table 3), which could be related to the high content of PUFAs (74%), mainly ALA (58%) and LA (16%). Both are valuable in diets since they are essential FAs that not synthesized in the body, so they must be obtained through food [32].

Another study reported a higher induction time (3.6 h), which decreased to 1.4 h in 6 months [13]. Unexpectedly, the latter value is closer to the present result (1.3 h). However, different conditions were used (110 °C, 10 L/h) in relation to the present study, in which a higher temperature (120 °C) and air flow rate (20 L/h) were used, which could have accelerated the oxidation process, explaining the lower induction period. Fortification with antioxidants or combining flaxseed oil with a more saturated oil could be strategies to achieve higher induction times [14]. Another approach to protect this oil from oxidation could be microencapsulation [47].

A higher *n*-6/*n*-3 ratio has been associated with the incidence of NCD [48]. However, flaxseed oil presented a low *n*-6/*n*-3 ratio (0.3, Table 3), which can counterbalance the harmful effects of higher dietary consumption of SFAs (8%) and LA (16%) in relation to ALA (58%).

MUFAs (18%) such as OA (18%) are recognized to reduce LDL levels, preventing atherosclerosis. Vegetable oils rich in unsaturated FA can easily suffer oxidation and produce toxic metabolites, especially with exposure to high temperatures. Nevertheless, antioxidants can improve oxidative stability [32].

Interestingly, another study reported a higher content of OA (23%), resulting in a lower PUFAs (66%) [49], which can be explained by different edaphoclimatic conditions or different origins of the seeds [37].

The vitamin E profile revealed α-tocotrienol and α-, γ-, and δ-tocopherols, totalling 444 mg/kg. This antioxidant appears to be the major contributor for oil protection since total phenolics (2.3 mg GAE/100 g) and total flavonoids (0.4 mg ECE/100 g) were low.

Another study with cold-pressed flaxseed oil found traces of α-tocopherol and contents of γ- and δ-tocopherols (35.3 mg/100 g and 0.2 mg/100 g, respectively), but did not identify α-tocotrienol, unlike the present data [49].

Although a high PUFA content (74%, Table 3) can present several health benefits, this oil presents low oxidative stability (1.3 h) and, consequently, low thermostability, which limits its use for cooking. If this oil presented higher contents of MUFAs (18%), its oxidative stability could be enhanced, resulting in better shelf-life since OA is known to promote stability in foodstuffs. However, its use in cooking has been recently recommended because of high lignin contents. The formation of oil blends with more SFA- and MUFA-rich oils can be proposed to increase stability [32,50,51].

Nevertheless, no products of primary oxidation (K_232nm_) and secondary oxidation (K_270nm_) were produced, and the peroxide value was low (2.4 meq O_2_/kg, Table 3). However, these analyses were performed after oil extraction. The shelf-life period should be monitored to evaluate if these values increase with time. In another study, higher K_232 nm_ and K_270nm_ values were reported for this oil (1.50 and 0.24, respectively), although the peroxide value was lower 1.85 meq O_2_/kg [52]. The use of amber containers for storage is recommended as light exposure can accelerate oxidation [50].

Therefore, this oil has the potential to be consumed raw, e.g., salad dressing, or used in the formulation of food supplements, contributing for the daily uptake of essential FAs, i.e., ALA (58%) and LA (16%), and antioxidants, i.e., vitamin E (444 mg/kg). Flaxseed oil’s dietary supplementation had a positive effect in reducing type II diabetes due to inflammation reduction and changes in gut microbiota composition [51]. However, due to its expensive price, this oil is restricted to a niche market, being considered a specialty oil; therefore, it could have applications as a dietary supplement or in the paint and varnish industry [46].

Herchi et al. reported that DPPH^∙^ inhibition decreased from 65% to 50% during the heating of flaxseed oil, which can be explained by the decrease in total phenolics from 84 to 60 GAE, mg/100 g, and total flavonoids from 18 to 12 luteolin equivalents, mg/100 g, which also happened for carotenoid and chlorophyll contents [52].

The following phenolic compounds were reported in flaxseed oil: secoisolariciresnol, ferulic acid and its methyl ester, coumaric acid methyl ester, diphyllin, pinoresinol, matairesinol, *p*-hydroxybenzoic acid, vanillin, and vanillic acid [53]. Although the oil presented low total phenolics (2.3 mg, GAE/100 g) and total flavonoids (0.4 mg, ECE/100 g) and antioxidant activity (FRAP assay: 96.0 μmol, FSE/100 g; DPPH^∙^ inhibition: 0.2 mg, TE/100 g), it should be noted that the oil is protected inside the seed, where it is not exposed to environmental stresses; therefore, higher contents were obtained for flaxseeds and flour as discussed before.

### 4.4. Future Research

Regarding future research, the bioactivity of the seed, flour, and oil extracts should be further investigated by employing cellular assays (e.g., anti-diabetic, neuroprotective, anti-inflammatory potential, anti-allergic properties, and anti-microbial activity).

More research is also recommended to evaluate the shelf-life period and optimal storage conditions for these products to ensure their stability over time. This could involve studying the impact of different environmental factors, such as temperature, light, and humidity, on the degradation of bioactive compounds. Exploring novel preservation techniques, such as encapsulation or freeze-drying, may also enhance the longevity of these products.

Additionally, the bioavailability and metabolism of bioactive compounds after digestion could be assessed by the in vitro simulations of gastrointestinal digestion, so that food matrix interactions or the role of the gut microbiota and the outcomes on human health are better understood and validated.

## 5. Conclusions

There is a growing demand for functional foods containing bioactive compounds, such as flaxseeds. This study evaluated the chemical composition of flaxseeds to assess their nutritional relevance, highlighting that cold pressing is an effective method for oil extraction, yielding high-quality oil as well as defatted flour as a by-product. These products contained vitamin E and phenolic compounds, which appear to be the main contributors to the antioxidant properties of the samples.

Cold-pressed flaxseed oil has potential as a raw consumable, such as in salad dressings or nutraceuticals, due to its essential fatty acids (in particular LA, and predominantly ALA) and vitamin E content (α-tocotrienol and α-, γ-, and δ-tocopherols). Given its likely high market price, it can be considered a specialty oil, with potential applications as a dietary supplement.

Flaxseed flour, the by-product of oil extraction, should be valued and incorporated into the food industry, as it was found to be a richer source of total minerals, high-quality gluten-free protein, and high-fibre compared to whole flaxseeds. This makes it a valuable ingredient that can provide various functional properties to food products, aligning with goals for food and nutritional security, as well as circular economy and sustainability.

Although flaxseed products can offer significant nutritional benefits, they cannot compensate for poor dietary habits, such as the excessive consumption of saturated fats. Future research should focus on investigating bioactivity through cellular assays, evaluating shelf-life and storage conditions, and assessing bioavailability and metabolism after digestion to better understand and validate the health impacts of these products.

## Figures and Tables

**Table 1:** Composition of whole flaxseeds and defatted flour.

Parameter	Seeds	Flour
Energy Value (kcal/100 g)	475 ± 1 ^a^	314 ± 6 ^b^
Energy Value (kJ/100 g)	1954 ± 5 ^a^	1304 ± 26 ^b^
Moisture (%)	8.42 ± 0.11 ^b^	9.78 ± 0.13 ^a^
Ash (%)	3.64 ± 0.02 ^b^	5.64 ± 0.02 ^a^
Total Protein (%)	16.07 ± 0.07 ^b^	28.08 ± 0.82 ^a^
Total Dietary Fibre (%)	33.73 ± 0.00 ^b^	35.61 ± 0.00 ^a^
Insoluble Fibre (%)	30.22 ± 0.00 ^a^	25.66 ± 0.60 ^b^
Soluble Fibre (%)	3.51 ± 0.00 ^b^	9.96 ± 0.43 ^a^
Remaining Carbohydrates (%)	0.03 ± 0.13 ^b^	11.56 ± 0.56 ^a^
Total Fat (%)	38.11 ± 0.04 ^a^	9.33 ± 0.14 ^b^
Fatty Acids (Relative %)		
C16:0 (Palmitic Acid)	5.06 ± 0.04 ^b^	6.30 ± 0.07 ^a^
C16:1*c* (Palmitoleic Acid)	0.06 ± 0.00 ^b^	0.07 ± 0.00 ^a^
C17:0 (Margaric Acid)	0.06 ± 0.00 ^a^	0.05 ± 0.00 ^b^
C18:0 (Stearic Acid)	3.35 ± 0.04 ^a^	3.21 ± 0.15 ^a^
C18:1n9*c* (Oleic Acid)	18.02 ± 0.09 ^b^	18.28 ± 0.07 ^a^
C18:2n6*c* (Linoleic Acid)	16.58 ± 0.08 ^b^	17.07 ± 0.16 ^a^
C18:3n3*c* (α-Linolenic Acid)	56.75 ± 0.19 ^a^	54.88 ± 0.28 ^b^
C20:0 (Arachidic Acid)	0.11 ± 0.00 ^b^	0.13 ± 0.00 ^a^
∑SFA (Saturated Fatty Acids)	8.59 ± 0.08 ^b^	9.70 ± 0.15 ^a^
∑MUFA (Monounsaturated Fatty Acids)	18.08 ± 0.09 ^b^	18.35 ± 0.07 ^a^
∑PUFA (Polyunsaturated Fatty Acids)	73.33 ± 0.13 ^a^	71.95 ± 0.16 ^b^
*n*-6/*n*-3	0.29 ± 0.00 ^b^	0.31 ± 0.00 ^a^
Total Vitamin E (mg/kg)	214.68 ± 9.34 ^a^	48.49 ± 0.66 ^b^
α-Tocopherol	73.67 ± 2.01 ^a^	17.39 ± 0.14 ^b^
γ-Tocopherol	141.01 ± 7.49 ^a^	30.98 ± 0.53 ^b^
Total Amino Acids (mg/g)	194.17 ± 8.92 ^b^	314.18 ± 11.21 ^a^
Asp	19.31 ± 0.90 ^b^	31.31 ± 1.10 ^a^
Glu	39.99 ± 1.99 ^b^	64.94 ± 2.23 ^a^
Ser	10.10 ± 0.48 ^b^	16.14 ± 0.58 ^a^
Gln	1.01 ± 0.09 ^b^	2.03 ± 0.24 ^a^
* His	6.24 ± 0.27 ^b^	9.56 ± 0.27 ^a^
Gly	12.42 ± 1.59 ^b^	22.30 ± 0.92 ^a^
* Thr	7.75 ± 0.35 ^b^	12.15 ± 0.34 ^a^
Arg	22.70 ± 1.18 ^b^	36.59 ± 1.34 ^a^
Ala	9.20 ± 0.43 ^b^	14.90 ± 0.54 ^a^
Tyr	4.33 ± 0.19 ^b^	6.43 ± 0.25 ^a^
* Val	9.55 ± 0.51 ^b^	15.41 ± 0.51 ^a^
* Met	2.80 ± 0.39 ^b^	3.95 ± 0.16 ^a^
* Trp	1.39 ± 0.03 ^a^	1.47 ± 0.25 ^a^
* Phe	9.75 ± 0.22 ^b^	15.87 ± 1.87 ^a^
* Ile	8.54 ± 0.48 ^b^	12.94 ± 0.47 ^a^
* Leu	11.83 ± 0.41 ^b^	18.76 ± 0.77 ^a^
* Lys	9.12 ± 0.92 ^b^	15.98 ± 0.70 ^a^
Hyp	1.50 ± 0.02 ^b^	2.47 ± 0.07 ^a^
Pro	6.63 ± 0.16 ^b^	10.99 ± 0.30 ^a^
Total Phenolics (mg GAE/100 g)	142.5 ± 7.2 ^b^	204.9 ± 19.9 ^a^
Total Flavonoids (mg EE/100 g)	53.9 ± 2.4 ^b^	78.2 ± 4.5 ^a^
FRAP (mmol FSE/100 g)	8.5 ± 0.4 ^b^	14.3 ± 1.9 ^a^
DPPH^∙^ Inhibition (mg TE/100 g)	57.2 ± 11.0 ^b^	233.3 ± 22.6 ^a^

* Essential amino acids. Results in fresh weight. Values presented as mean ± standard deviation (*n* = 3). Different small-case letters in the same row denote significant differences (*p* < 0.05). GAEs, gallic acid equivalents; EEs, epicatechin equivalents; FRAP, ferric reduction antioxidant power; FSEs, ferrous sulphate equivalents; DPPH^•^, 2,2-diphenyl-1-picrylhydrazyl; TE, Trolox equivalents.

**Table 2:** Protein quality of whole flaxseeds and defatted flour.

EAA	AA Estimates for Adults * (mg/g Protein)	Seeds (mg/g Protein)	Flour (mg/g Protein)	Seed AACS (%)	Flour AACS (%)
His	15	38.81 ± 1.68 ^a^	34.04 ± 0.98 ^b^	258.72 ± 11.19 ^A^	226.90 ± 6.53 ^B^
Ile	30	53.14 ± 2.98 ^a^	46.08 ± 1.67 ^b^	177.15 ± 9.95 ^A^	153.59 ± 5.56 ^B^
Leu	59	73.63 ± 2.53 ^a^	66.82 ± 2.74 ^b^	124.79 ± 4.28 ^A^	113.25 ± 4.64 ^B^
Lys	45	56.75 ± 5.75 ^a^	56.89 ± 2.49 ^a^	126.11 ± 12.78 ^A^	126.43 ± 5.54 ^A^
Met	16	17.44 ± 2.42 ^a^	14.07 ± 0.56 ^a^	109.03 ± 15.15 ^A^	87.96 ± 3.47 ^A^
Phe + Tyr	38	87.67 ± 2.54 ^a^	79.44 ± 7.51 ^a^	230.70 ± 6.69 ^A^	209.04 ± 19.77 ^A^
Thr	23	48.20 ± 2.18 ^a^	43.26 ± 1.21 ^b^	209.56 ± 9.46 ^A^	188.10 ± 5.28 ^B^
Trp	6	8.68 ± 0.16 ^a^	5.25 ± 0.91 ^b^	144.67 ± 2.66 ^A^	87.50 ± 15.10 ^B^
Val	39	59.42 ± 3.15 ^a^	54.86 ± 1.81 ^a^	152.36 ± 8.08 ^A^	140.68 ± 4.63 ^A^
LAA (%)	-	-	-	Met 109.03 ± 15.15 ^A^	Trp 87.50 ± 15.10 ^A^
EAAI (%)	-	129.45 ± 6.26 ^a^	111.83 ± 2.23 ^b^	-	-

* AA estimates for adults according to WHO/FAO/UNU (2007). Values presented as mean ± standard deviation (*n* = 3). Different small-case letters in the same row denote significant differences (*p* < 0.05). Different capital letters in the same row denote significant differences (*p* < 0.05). AA, amino acids; AACS, amino acid chemical score; EAA, essential amino acid; LAA, limiting amino acid; EAAI, essential amino acid index.

**Table 3:** Composition of cold-pressed flaxseed oil.

Parameter	Oil
Oxidative Stability (h)	1.3 ± 0.1
Peroxide Value (meq O_2_/kg)	2.4 ± 0.0
K_232nm_	0.016 ± 0.001
K_270nm_	0.0500 ± 0.0005
Chromatic coordinates (x, y)	(0.4749, 0.4801)
Transparency (%)	65.8
Dominant wavelength (nm)	577.8
Purity	88.0
Fatty Acids (Relative %)	
C16:0 (Palmitic Acid)	4.81 ± 0.02
C18:0 (Stearic Acid)	3.18 ± 0.09
C18:1n9*c* (Oleic Acid)	18.47 ± 0.12
C18:2n6*c* (Linoleic Acid)	16.03 ± 0.08
C18:3n3*c* (α-Linolenic Acid)	57.51 ± 0.13
∑SFA (Saturated Fatty Acids)	7.99 ± 0.11
∑MUFA (Monounsaturated Fatty Acids)	18.47 ± 0.12
∑PUFA (Polyunsaturated Fatty Acids)	73.54 ± 0.21
*n*-6/*n*-3	0.28 ± 0.00
Total Vitamin E (mg/kg)	443.91 ± 6.23
α-Tocopherol	3.96 ± 0.10
α-Tocotrienol	3.68 ± 0.05
γ-Tocopherol	431.72 ± 6.39
δ-Tocopherol	4.55 ± 0.32
Total Phenolics (mg GAE/100 g)	2.3 ± 0.2
Total Flavonoids (mg EE/100 g)	0.4 ± 0.1
FRAP (μmol FSE/100 g)	96.0 ± 1.8
DPPH^∙^ Inhibition (mg TE/100 g)	0.2 ± 0.1

Results in fresh weight. Values presented as mean ± standard deviation (*n* = 3). K, extinction coefficient; GAEs, gallic acid equivalents; EEs, epicatechin equivalents; FRAP, ferric reduction antioxidant power; FSEs, ferrous sulphate equivalents; DPPH^∙^, 2,2-diphenyl-1-picrylhydrazyl; TE, Trolox equivalents.

## Data Availability

All data are contained within the article.
